# Organic Matter Type Defines the Composition of Active Microbial Communities Originating From Anoxic Baltic Sea Sediments

**DOI:** 10.3389/fmicb.2021.628301

**Published:** 2021-05-05

**Authors:** Saara Suominen, Daan M. van Vliet, Irene Sánchez-Andrea, Marcel T. J. van der Meer, Jaap S. Sinninghe Damsté, Laura Villanueva

**Affiliations:** ^1^Department of Marine Microbiology and Biogeochemistry, NIOZ Royal Netherlands Institute for Sea Research, Den Burg, Netherlands; ^2^Wageningen Food and Biobased Research (WFBR), Bornse Weilanden 9, Wageningen, Netherlands; ^3^Laboratory of Microbiology, Wageningen University, Wageningen, Netherlands; ^4^Department of Earth Sciences, Faculty of Geosciences, Utrecht University, Utrecht, Netherlands

**Keywords:** sediment, microbial ecology, DNA stable isotope probing, organic matter, acetate, fermentation, Chloroflexi, Bathyarchaeota

## Abstract

Carbon cycling in anoxic marine sediments is dependent on uncultured microbial communities. Niches of heterotrophic microorganisms are defined by organic matter (OM) type and the different phases in OM degradation. We investigated how OM type defines microbial communities originating from organic-rich, anoxic sediments from the Baltic Sea. We compared changes in the sediment microbial community, after incubation with different stable isotope labeled OM types [i.e., particulate algal organic matter (PAOM), protein, and acetate], by using DNA stable isotope probing (DNA-SIP). Incorporation of ^13^C and/or ^15^N label was predominantly detected in members of the phyla *Planctomycetes* and *Chloroflexi*, which also formed the majority (>50%) of the original sediment community. While these phylum-level lineages incorporated label from all OM types, phylogenetic analyses revealed a niche separation at the order level. Members of the MSBL9 (*Planctomycetes*), the *Anaerolineales* (*Chloroflexi*), and the class *Bathyarchaeota*, were identified as initial degraders of carbohydrate-rich OM, while other uncultured orders, like the CCM11a and *Phycisphaerales* (*Planctomycetes*), *Dehalococcoidia*, and JG30-KF-CM66 (*Chloroflexi*), incorporated label also from protein and acetate. Our study highlights the importance of initial fermentation of complex carbon pools in shaping anoxic sediment microbial communities and reveals niche specialization at the order level for the most important initial degraders in anoxic sediments.

## Introduction

Marine sediments are a living ecosystem (Parkes et al., 2014), containing roughly as many cells as found in the oceans and soils (Kallmeyer et al., 2012). Microbial communities living in marine sediments are responsible for driving the sedimentary biogeochemical cycles (Jørgensen, 2006), including the cycling of a large organic matter (OM) reservoir (Mackenzie et al., 2004). Most members of these microbial communities remain uncultured and therefore the details of their metabolic activity and the physicochemical or biological limits that delineate their activity and survival are not well understood (Lloyd et al., 2018). Thus, understanding the dynamics in microbial communities reliant on organic carbon is required to better define their role in carbon cycling in sediments.

The niches of microbial communities in sediments are likely defined by a multitude of factors, including the redox state of the sediment, the composition of OM, and its phase of degradation (Arndt et al., 2013). The identity and activity of the organisms responsible for the initial phases of OM degradation in sediments remain especially poorly characterized (Arnosti, 2011; Arndt et al., 2013). Essentially, OM degradation in anoxic environments proceeds in a stepwise manner, where the interaction of several microbial groups is necessary for the full degradation of complex organic compounds (Jørgensen, 2006; Schink and Stams, 2012; Arndt et al., 2013). Initial degraders produce and export extracellular enzymes for the degradation of complex organic polymers such as carbohydrates and proteins to oligomers or monomers of sugars and amino acids (Arnosti, 2011). These monomers are fermented to intermediate compounds like volatile fatty acids (VFAs) and alcohols. Secondary fermenters utilize the intermediate compounds and produce mainly acetate and hydrogen in the acetogenic degradation phase (Arndt et al., 2013). Finally, in the terminal stages of OM degradation acetate or hydrogen can be oxidized by microorganisms utilizing terminal electron acceptors (TEAs) like sulfate or utilized by methanogens to produce methane (Arndt et al., 2013).

The OM pool in sediments is composed of thousands of different molecules with various sizes and structural components (Burdige, 2007; Arnosti, 2011). When considering the thermodynamics of OM oxidation, different organic molecules have different energetic potential and availability to microbial utilization especially in anoxic conditions (Wakeham and Canuel, 2006; Zonneveld et al., 2010; Boye et al., 2017) and their degradation requires the production of a suite of specialized enzymes that is energetically expensive (Arnosti, 2011; Orsi et al., 2018). Therefore, it is likely that the available substrate type plays a major role in shaping the microbial community responsible for initial OM hydrolysis and degradation (Mayor et al., 2012; Mahmoudi et al., 2017).

Novel “omics” methodologies provide valuable insight into the genetic potential of microbes in the environment. Sediment microbial communities have been extensively studied with these methods (e.g., Biddle et al., 2008; Kirchman et al., 2014; Wrighton et al., 2014; Baker et al., 2015; Anantharaman et al., 2016; Thureborn et al., 2016; Marshall et al., 2017; Zinke et al., 2017, 2019; Orsi et al., 2018; Rasigraf et al., 2019). However, methods that provide insight into microbial substrate assimilation are required to understand the role that these microorganisms play in relation to the substrates available to them (Middelburg, 2014). DNA stable isotope probing (DNA-SIP) provides the opportunity to follow the flow of defined substrates through complex processes like those involved in anaerobic OM degradation (Radajewski et al., 2000). In combination with next generation sequencing methods, DNA-SIP can give a detailed view on carbon flow across microbial communities (Bell et al., 2011; Hungate et al., 2015; Orsi et al., 2016, 2019; Pepe-Ranney et al., 2016). Previously, SIP studies have been made in sediments to study the microorganisms responsible for heterotrophic or autotrophic metabolism (Coskun et al., 2018; Orsi et al., 2019), the degradation of phytoplankton biomass in surface sediments (Gihring et al., 2009; Graue et al., 2012a; Müller et al., 2018), and the degradation of simple sugars and other intermediates (Webster et al., 2010; Graue et al., 2012b; Vandieken and Thamdrup, 2013; Na et al., 2015), while few studies have directly compared the microbial communities degrading different complex OM pools in marine sediments (Seyler et al., 2014).

The aim of this study was to understand how, and to what extent, the composition of microbial communities is affected by supplementing different forms of OM under sulfate-depleted conditions. The microbial communities studied here originate from OM-rich sediments below oxygen-depleted waters in the Gotland basin in the Baltic Sea. Baltic Sea sediment microbial communities have been extensively studied across different environmental gradients (Edlund et al., 2006; Thureborn et al., 2016; Marshall et al., 2017; Klier et al., 2018; Bird et al., 2019; Rasigraf et al., 2019; Zinke et al., 2019; Jørgensen et al., 2020), and the sedimentary OM content has been found to be one of the main defining factors of microbial community composition (Edlund et al., 2006; Rasigraf et al., 2019; Zinke et al., 2019). This makes them an ideal model system to test the effects of OM type on the microbial community. To understand how OM type determines the microbial community composition and how the niches of heterotrophic anoxic sediment microbial communities are defined, we compare the community assimilating both complex and intermediate OM substrates across different degradation phases.

## Materials and Methods

### Sediment Collection and Storage

Sediment cores used as inocula in the experiment were collected from the permanently anoxic Gotland Deep in the Baltic Sea (57° 12.702'N, 19° 57.000'E) at 220 m water depth with R/V Pelagia (cruise number 64PE410) in May 2016. Cores were collected with a multicorer resulting in 12 cores of 30 cm length. One core was immediately sliced in 1 cm horizons and stored at −80°C for DNA analysis. Samples of sediment horizons were also freeze-dried and used to determine the total organic carbon (TOC) concentration. Porewater samples were collected by slicing cores under anoxic conditions inside a glove bag under N_2_ overpressure each 1 cm for the first 30 cm. The retrieved sediment was centrifuged using 50 ml Greiner tubes at 3,000 rpm for 15–30 min depending on the amount of water recovered. Porewater was collected and stored at −80°C for VFA analysis. The remaining cores were stored at 4°C for 18 months until incubations started. Due to the long storage period, the microbial community composition of the inocula is not considered to represent *in situ* conditions. However, we considered it as still containing organisms that would be reliant on organic matter and could be enriched with substrate amendment. The microbial community of the slurries was analyzed from *in situ* sediment samples as well as from sediment samples after storage at time zero (T0) of the incubations to estimate how the storage affected the incubation results.

### Substrate Preparation

Two substrates (particulate organic matter and protein extract) were prepared from biomass obtained from cultures of the eustigmatophyte algae *Nannochloropsis oculata* strain CCMP 2195. *N. oculata* was grown in f/2 media (Guillard and Ryther, 1962; Guillard, 1975) supplemented with 40 mM NaHCO_3_ (Supplementary Table S2). For labeled incubations, 30% of the NaHCO_3_ was replaced by ^13^C-NaHCO_3_ (Cambridge Isotope Laboratories, Tewksbury, MA, United States), and 10% of NaNO_3_ (at a concentration of ca. 0.9 mM) was replaced with ^15^N-NaNO_3_ (Sigma Aldrich, St. Louis, MO, United States). The algal cultivation was carried out in 15°C with a light dark cycle of 12 h for 2 weeks. Biomass was collected with a continuous centrifuge at 10,000 × *g*, and stored at −20°C. The pellet was resuspended in growth media (1.25 g wet weight/20 ml) and cells were broken by high-pressure homogenization (SPCH-10, Stansted Fluid Power ltd., Essex, United Kingdom) with 6 rounds at 125 MPa (18130 Psi). The cell debris was then centrifuged (5,000 × *g*, 30 min, 4°C), the supernatant discarded and the remaining insoluble material freeze-dried and used as “particulate algal organic matter” (PAOM) substrate for the incubations.

Protein was extracted from pelleted biomass (i.e., before breaking cells) with a Trizol-based method (Kirkland et al., 2006), and subsequently freeze-dried and used as the “protein” substrate for the incubations. The percentage of ^13^C and ^15^N of the labeled substrates was measured by EA-IRMS (Flash 2000 EA with a DeltaV Advantage; Thermo Fisher Scientific, Bremen, Germany) equipped with a NC/NCS 3 m column.

### Sediment Slurries

Trace sulfide concentrations were measured in the porewater after the storage period, indicating that the center of the cores remained anoxic throughout this period. Sediment slurries were prepared inside an anoxic glove bag with a 100% N_2_ atmosphere. All materials used were autoclaved and kept in anoxic conditions one night prior to the slurry setup. The 20–30 cm horizon of four cores was obtained inside an anoxic glove bag under N_2_ overpressure, stored in oxygen-tight boxes and transferred to another anoxic glove bag, where a total of 1.2 kg of sediment was thoroughly mixed with 2.5 L of medium composed of anoxic, sulfate-free, brackish water media (Supplementary Table S3), supplemented with 0.5 mg/L resazurin and 150 μM sodium sulfide and buffered with 3 mM NaHCO_3_. Aliquots of 60 ml slurry were distributed into 120 ml pressure bottles and closed inside the glove bag. To ensure that the slurries were as homogeneous as possible, the total slurry was thoroughly mixed throughout the process of aliquoting. The headspace of each bottle was exchanged with N_2_:CO_2_ (80:20) through a sterile 0.2 μm syringe filter with an automatic gas exchanger (GR instruments, Utrecht, The Netherlands). Slurries were preincubated at 15°C in the dark for 2 weeks to deplete the labile OM that may have become available during the sediment mixing because the study objective was to accentuate the effect of the added substrates.

After 2 weeks, the incubations were started at 15°C in the dark with the addition of substrates inside a glove box. Sediments were amended with either 1.67 mg/ml of PAOM, 1.67 mg/ml of protein (prepared as described above), or 0.7 mg/ml of acetate (^13^C-labeled at C-1, Sigma Aldrich, St. Louis, MO) in both labeled and unlabeled form. In contrast to the complex substrates, the commercially acquired acetate was therefore 50% ^13^C labeled. The complex substrates (PAOM and protein) amounted to ca. 50% of the sedimentary TOC. Based on the measured fraction of carbon in the substrate, a total of 17 mM carbon was added to the PAOM incubations and 51 mM to the protein incubations. Acetate was added at 10 mM following Na et al. (2015). All substrates were added, both in labeled and unlabeled form, to compare the change in the density distribution of specific 16S rRNA gene sequences between unlabeled and labeled conditions. For each substrate, 3–6 slurries were prepared and one slurry was used at each time point for DNA analysis. Killed controls were prepared by autoclaving slurries. In addition to the substrate-amended slurries, unamended slurries, and killed slurries with and without substrate addition were used as controls.

Slurries were sampled weekly at first (0, 7, 14, 21, 28, and 35 days) and then biweekly (49 and 63 days) to determine H_2_, CH_4_, HS, ^13^C-DIC, and acetate concentrations as described in the following section. At days 0, 7, 21, and 63, one slurry of each substrate type was used for microbial community analysis. Slurries were collected in 50 ml Greiner tubes and centrifuged for 10 min 4°C at 4,000 rpm. The sediment was stored at −80°C before nucleic acid extraction, and the remaining supernatant was filtered through a 0.2 μm Acrodisc syringe filter with a Supor membrane (Pall Corporation, New York, NY) and frozen at −20°C for the analysis of NH_4_^+^concentrations.

### Analysis of Nutrients and Metabolites

H_2_ and CH_4_ concentrations were measured directly from the headspace of the slurries. For all samples, the volume taken was replaced by sterile N_2_ gas. A sample of 2 ml was taken from the liquid phase of mixed sediment slurries for acetate measurements. This sample was centrifuged for 10 min at 12,000 × *g* and supernatant was collected and frozen at −20°C until analysis. A liquid sample of 0.5 ml was collected from the liquid phase for ^13^C-DIC measurements in glass exetainers. The exetainer was filled with saturated salt solution (41 g/L NaCl) to exclude any headspace and poisoned with 25 μl of saturated HgCl_2_ solution before storing upside down at 4°C until analysis. Samples of 0.5 ml were collected for the analysis of sulfide concentrations. The samples were immediately added to an Eppendorf tube containing 25 μl 5% ZnCl solution, and measured on the same day with the methylene blue method (Cline, 1969).

The TOC content (in %) of the sediments was measured using freeze-dried and acidified sediment samples with a Flash 2000 series Elemental Analyzer (Thermo Fisher Scientific, Waltham, MA, United States) equipped with a TCD detector. The concentrations of H_2_ and CH_4_ were measured from the headspace of slurries with a Global Analyzer Solution GC (Interscience, Woburn, MA, United States) equipped with a Carboxen 1010 pre-column and a mol sieve 5A as the main column and a pulsed discharge ionization detector. Porewater acetate concentrations from the sampling site were measured with two-dimensional ion chromatography mass spectrometry (Glombitza et al., 2014). Concentrations of acetate in slurries were measured with a Spectrasystems HPLC (Thermo Fisher Scientific, Waltham, MA, United States) equipped with a HiPlex-H column (Agilent, Santa Clara, CA) and a RI detector. The ^13^C content in dissolved inorganic carbon (DIC) was measured with a DeltaV Advantage coupled to a Gasbench II (Thermo Fisher Scientific, Waltham, MA, United States). NH^4+^ concentrations were analyzed using TRAACS Gas Segmented Continuous Flow Analyzer (Seal Analytical, United Kingdom) according to the method of Helder and De Vries (1979).

### DNA Extraction and 16S rRNA Gene Amplicon Sequencing of the Original Sediment

Original sediment samples sliced and stored at −80°C were analyzed to estimate *in situ* microbial community composition. DNA and RNA were extracted from 2 g of sediment from sediment depths of 1–2, 10–11, 20–21, and 25–26 cm using the RNA PowerSoil® Total RNA Isolation Kit with the RNA PowerSoil DNA Elution Accessory Kit (Mo Bio Laboratories Inc., Carlsbad, CA, United States) according to the manufacturer’s instructions. Nucleic acid concentrations were quantified fluorometrically with QubitTM RNA HS Assay kit/dsDNA HS assay kit (ThermoFischer Scientific, Waltham, MA, United States). The V4 region of the 16S SSU rRNA gene was amplified as described in Moore et al. (2015). Amplicons were combined at equal concentrations, concentrated with the MinElute PCR Purification Kit (QIAGEN, Hilden, Germany), and sequenced with 454 GS FLX (Roche) at Macrogen Inc. (Seoul, South Korea).

### DNA Extraction and Size Selection From Sediment Slurries

The DNA extractions from the sediment incubations were performed following the protocol of Griffiths et al. (2000). Briefly, 5 g (wet weight) of sediment were added to 50 ml tubes containing 5 g of 0.1 mm zirconia beads and 5 g of 1 mm zirconia beads (Biospec, Bartlesville, OK, United States) and mixed with 10 ml pH 8 phosphate buffered lysis buffer (240 mM Na2HPO4:NaH2PO4, 0,5% N-lauryl sarcosine) and 10 ml of phenol:chloroform:isoamyl alcohol (25:24:1). The mixture was bead-beaten two times for 30 s at 2.4 m/s using a Beadrupter elite (Omni International, Kennesaw, GA, United States). Then, 1.7 ml of salt solution (5 M NaCl) and 1.2 ml of extraction buffer (10% CTAB, 0.7 M NaCl) were added, vortexed, and the tube incubated for 1 min on ice. The samples were centrifuged for 5 min at 4°C with 2,500 × *g* and the aqueous layer (~10 ml) was transferred to a new tube and kept on ice. To increase the DNA yield, the sample was back extracted by adding 10 ml of lysis buffer and 1.7 ml of salt solution again to the bead containing tube. Bead beating and centrifugation were repeated and both aqueous phases were combined. Subsequently, the sample was washed by adding an equal volume of chloroform:isoamyl alcohol (24:1); after centrifugation the aqueous phase (~20 ml) was again transferred to a new tube. Next, twice the volume of precipitation buffer (30% PEG 6000 and 1.6 M NaCl) was added to the sample and left for precipitation at room temperature for 2 h, followed by centrifugation for 30 min at 2,500 × *g*. The supernatant was discarded, and the pellets were washed with 2 ml ice cold ethanol (70%) and collected by centrifuging 14,800 × *g* for 10 min at 4°C. Pellets were air dried and finally eluted in 500 μl of nuclease-free water. DNA concentrations were quantified spectrophotometrically (Nanodrop, Thermo Fischer Scientific, Waltham, MA, United States) or fluorometrically with QubitTM dsDNA HS assay kit (Thermo Fischer Scientific, Waltham, MA, United States). The size range of about >1 kb DNA was selected by gel electrophoresis for 20 min at 70 V and 4°C (Buckley, personal correspondence) using low melting point agarose (Thermo Scientific, Waltham, MA, United States) at 0.8% in TAE buffer. Target DNA was cut out of gel and DNA was purified by β-agarase extraction (New England Biolabs, Ipswich, MA, United States), according to the manufacturer’s instructions, with home-made extraction buffer (100 mM bisTris pH 6.5, 10 mM EDTA; Moore et al., 2002).

### Ultracentrifugation of DNA and Illumina Sequencing of the 16S rRNA Gene

Density gradient centrifugation was performed with CsCl gradients as described in Dunford and Neufeld (2010). A final density 1.725 g/ml of the CsCl solution with 1 μg of DNA from each sample was centrifuged for 60 h at 44,000 rpm (177000 × gav) at 20°C using a Vti 65.2 rotor (Beckmann Coulter, Brea, CA, United States) and 5.1 ml QuickSeal Polyallomer tubes (Beckman Coulter, Brea, CA, United States). Each sample was fractionated to 12 equal fractions, and the density of each fraction was checked using 10 μl of sample with a digital refractometer (AR2000 Reichert Technologies, Buffalo, NY, United States). Buoyant density (BD) was estimated based on density measurements and refraction index (RI) measurements made on a standard curve using serial dilutions of a CsCl solution (*R* = 0.99, *p* << 0.001). The final formula used was *BD* = 10.302*RI-12.747. Two volumes of PEG solution (30% PEG6000, 1.6 M NaCl) and 20 μg of polyacrylamide as a carrier were added to precipitate DNA. The mixture was incubated at room temperature for 2 h and the DNA was pelleted by centrifugation at 13,000 × *g* at 4°C for 30 min. The resulting pellets were washed with 70% ethanol, air-dried and resuspended in PCR-grade water.

The V4 region of the 16S SSU rRNA gene was amplified from DNA samples from all collected fractions with forward primer 515F-Y and reverse primer 806RB (Caporaso et al., 2011; Apprill et al., 2015; Parada et al., 2016). The PCR reactions were made as described in Suominen et al. (2019). After combining samples at equal concentrations, the total sample was concentrated with the MinElute PCR Purification Kit (QIAGEN, Hilden, Germany), and sequenced at the University of Utrecht (Netherlands) on an Illumina Miseq platform as paired-end reads of 350 bp. Sequences are submitted to the sequence read archive under the bioproject number PRJNA702135.

### 16S rRNA Gene Amplicon Data Analysis

16S rRNA gene amplicon data from original sediments were preprocessed with qiime as described previously (Besseling et al., 2018). Briefly, after demultiplexing, sequences were quality-filtered with a minimum quality score of 25 and length between 250 and 350, and a maximum of two errors in the barcode sequence were allowed. 16S rRNA gene amplicon data from sediment slurries was analyzed with the pipeline Cascabel (Asbun et al., 2019) as described previously with modifications (Suominen et al., 2019). For both datasets, operational taxonomic units (OTUs) were picked with swarm (Mahé et al., 2015) using pick_otus.py of QIIME (Caporaso et al., 2010) with default parameters. The longest sequence of each OTU cluster was picked as representative. Singletons were removed prior to analysis. The taxonomy of the representative sequences was assigned using usearch_global of Vsearch (Rognes et al., 2016) against the SILVA database release 132 (Quast et al., 2013). All further analyses were carried out in the R packages Phyloseq (McMurdie and Holmes, 2013), HTSSIP (Youngblut et al., 2018a), and vegan (Oksanen et al., 2018). OTUs with a lower relative abundance than 0.005% were removed prior to analysis. In addition, OTUs taxonomically related to Cyanobacteria were also removed, as these could not be separated from DNA originating from the added substrate.

### Assessment of Labeled OTUs

In order to determine community members that were likely to be active PAOM and protein degraders, we used the high-resolution DNA-SIP (HRSIP; Pepe-Ranney et al., 2016; Youngblut et al., 2018a) analysis method. Isopycnic centrifugation allows the separation of DNA fragments based on their density, and therefore directly the amount of labeled atoms that are in the molecules. However, due to natural density variation because of nucleotide differences, and naturally occurring small amounts of heavy isotopes, the separation of DNA based on BD alone is not sufficient. Instead the density of a molecule needs to be compared to its native density in an unlabeled form (i.e., control incubations). HRSIP is a method, where the density differences between the labeled and native forms of specific genetic codes are compared in a statistically significant manner. To this end, we compared differences in DNA (represented by OTU distributions) across the density gradients in ^13^C/^15^N labeled complex substrate incubations and ^13^C labeled acetate incubations to the corresponding distributions in control incubations for each substrate.

Operational taxonomic units were considered labeled if they satisfied two criteria; (1) they were significantly more abundant in the heavy fractions of the DNA obtained from ^13^C/^15^N substrate incubations than in the heavy fractions of the DNA of control incubations; and additionally (2) they were not significantly more abundant in the light fractions of the DNA obtained from ^13^C/^15^N substrate incubations compared to the light fractions of the DNA of control incubations. The second part of the analysis was added as an additional control step, to rule out OTUs that had a significantly higher abundance throughout the gradient in the labeled incubations compared to control incubations, due to slight differences in the total community composition of the two complex substrate incubations.

In more detail, OTUs that had a significantly higher relative abundance in either the heavy fractions or the light fractions of DNA extracted from incubations with labeled substrates (^13^C/^15^N) compared to DNA obtained from control incubations were evaluated with a Deseq2 analysis (Supplementary Figures S5, S6). In the HRSIP method, a log_2_ fold change was calculated for each OTU, and its significance was evaluated with a one-sided Wald test, and *p*-values were adjusted for multiple comparisons with the Benjamini-Hochberg method. OTUs that were not detected in both the labeled and control incubations could therefore not be analyzed. We defined the heavy fractions as fractions with a density higher than 1.70 g/ml. A 10% false discovery rate was used as a threshold for the adjusted *p*-values to denote significance. Calculations were done using the HRSIP function of the HTSSIP R package (Youngblut et al., 2018a). Only OTUs that satisfied both requirements outlined above were considered as labeled.

For the phylogenetic analysis of labeled OTUs, sequences were first aligned with MAFFT (v. 7.310, Katoh and Standley, 2013) and trimmed with TRIMAL (v. 1.2rev59, Capella-Gutierrez et al., 2009) with the automatic method selection. A tree was calculated from trimmed sequences with IQ-TREE using automatic model selection and ultrafast bootstrap approximation (v. 1.6.7, Nguyen et al., 2015; Hoang et al., 2017; Kalyaanamoorthy et al., 2017). The final model selected by IQ-TREE to infer phylogeny was SYM+R10.

## Results

### Incubation Set Up and Physicochemical Conditions in the Sediment Slurries

At our sampling station in the permanently anoxic Gotland Deep of the Baltic Sea, the sedimentary ammonium concentration increased steadily with depth from 8.3 μM at 0.5 cm to 88.2 μM at 30 cm (Supplementary Figure S1). Sulfide concentrations reached a maximum of 85.2 μM at 26 cm depth and decreased below this. The TOC content was 3.9 ± 0.3%, and the acetate concentration was 5.9 ± 1.4 μM at 20–30 cm. Sediments from the bottom of the sulfate-reducing zone, located in the 20–30 cm horizon below sea floor (Piker et al., 1998; Jørgensen et al., 2020), and with interesting target groups based on 16S rRNA amplicon sequencing, were selected as inocula and slurries were prepared without the addition of sulfate in order to induce highly reduced conditions (Supplementary Table S3).

Sediment slurries were amended with different OM types to initiate incubations. PAOM (water-insoluble membrane elements) was obtained from *N. oculata* biomass and represents less labile cell parts like lipids and complex polysaccharides (Scholz et al., 2014), which are selectively preserved in deeper anoxic sediment layers (Burdige, 2007). The C and N content of PAOM was 12 and 1%, respectively. The low nitrogen content of the PAOM reveals that proteins did not form a major part of PAOM. Proteins were extracted from the same algal biomass as the second complex substrate. They can make up to half of the cell material of phytoplankton (Hulatt et al., 2017). The C and N content of the protein substrate was 37 and 14%, respectively. Incubations with acetate were also conducted to determine those microbes reliant on this main intermediate compound in the OM degradation process.

^13^C‐ and ^15^N-labeled substrates were prepared by growing an *N. oculata* culture with labeled bicarbonate and nitrate. With this preparation method all major types in the substrate (i.e., proteins, lipids, and carbohydrates) should be evenly labeled. The ultimate degree of labeling was 15% ^13^C and 9% of ^15^N for PAOM, and 18% ^13^C and 7% ^15^N for the protein fraction. This degree of labeling is different from other SIP experiments with complex phytoplankton-derived organic matter where they used commercially available, fully ^13^C-labeled Spirulina, a freshwater cyanobacterium (Graue et al., 2012a; Müller et al., 2018). We used partially labeled complex organic matter to prevent “overlabeling” (i.e., everything is labeled at the end of the experiment) in the long-term slurry incubations (cf. Middelburg, 2014). In our DNA-HRSIP approach, the organisms, which are identified from incorporating the partially labeled substrate (reflected by their labeled 16S rRNA) can thus be considered to represent some of the major degraders of complex organic matter.

**Figure 1:**
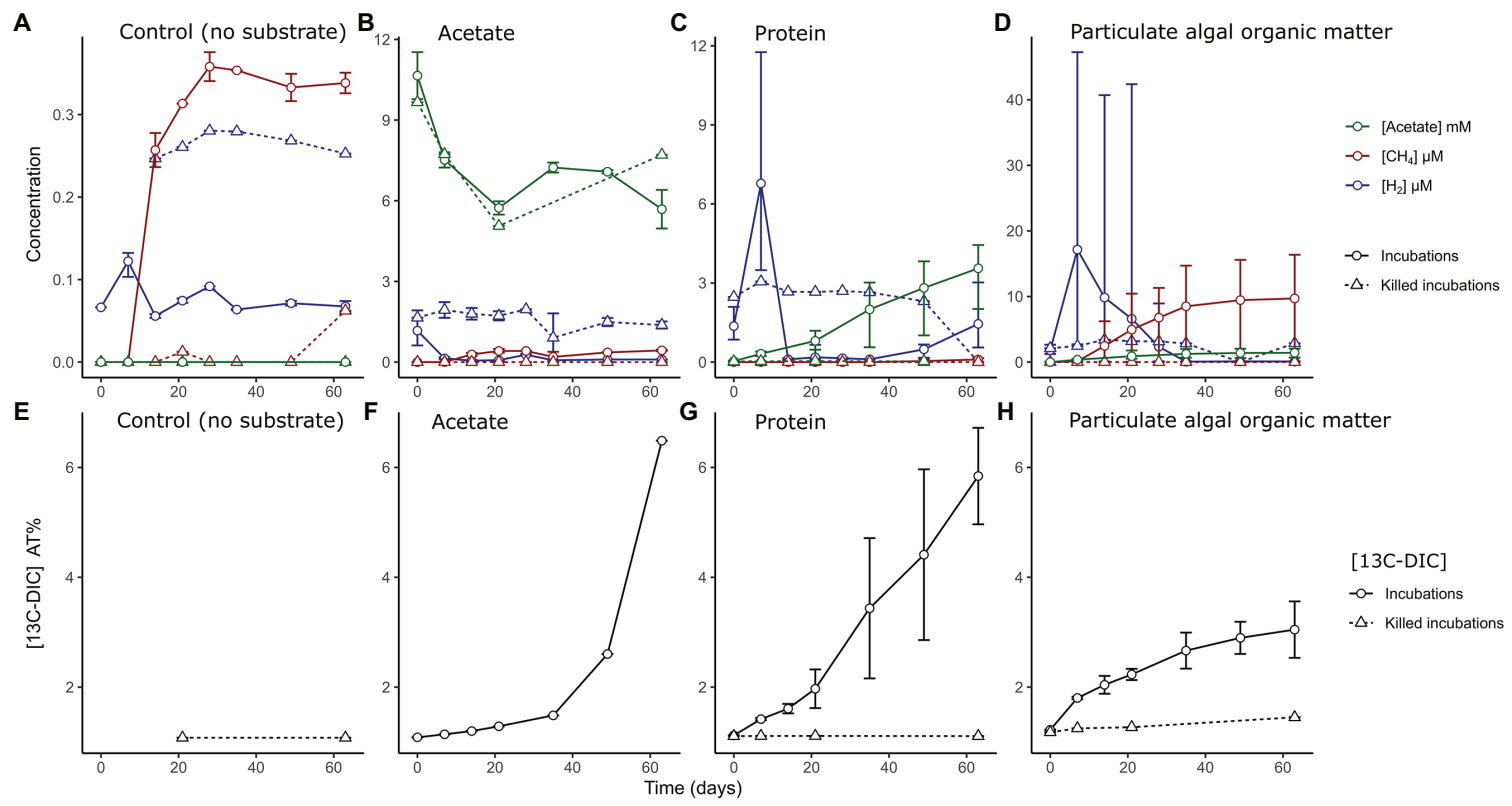
**(A–D)** Measured concentrations of hydrogen (blue, μM), methane (red, μM), and acetate (green, mM) in sediment slurries in control incubations without added substrate and in incubations amended with different organic matter types as indicated in the text. Measurements from incubations with both labeled and unlabeled substrates are combined. Note the different ranges of the y-axis. **(E–H)** Measured accumulation of ^13^C isotope fraction in dissolved inorganic carbon (DIC) as percentage in the different incubations and heat-killed controls. Solid lined represent live incubations, while dashed lines represent measurements from abiotic (killed) controls. Error bars depict the range of (*n* = 2–6) values.

Microbial activity was followed in the slurries by measuring the H_2_ and CH_4_ concentrations in the headspace, and acetate, sulfide, and ammonium concentrations as well as the ^13^C content of dissolved inorganic carbon (^13^C_DIC_) in the liquid phase (Figure 1; Supplementary Figure S1). In control slurries with no substrate amendment, we measured lower concentrations of intermediate metabolites (H_2_, CH_4_ or acetate; Figure 1A) when compared to OM-amended slurries (Figures 1B–D). H_2_ was utilized (0.07 ± 0.01 μM at 14–63 days) compared to killed slurries (0.26 ± 0.01 μM) and a minor amount of methane was produced after 2 weeks incubation time, reaching maximum concentrations at 4 weeks (0.4 μM). For the remaining incubation time, all measured values stayed stable, showing no clear signs of activity, other than a minor increase in the ammonium concentration (105 ± 64 μM at day 63; Supplementary Figure S2).

In slurries amended with acetate, H_2_ was quickly utilized from the headspace of live incubations, while killed controls contained trace H_2_ concentrations in the headspace throughout the incubations (1.7 ± 0.9 μM). The concentration of acetate decreased in both live and killed slurries until day 20 (Figure 1B). The ^13^C_DIC_ measurements indicated that ^13^C acetate was converted into DIC to a large extent only after >28 days of incubation (Figure 1F).

In slurries amended with protein, we detected an initial phase with a net production of H_2_ at day 7 (6.8 ± 2.7 μM); H_2_ became subsequently depleted by day 14 (Figure 1C). Acetate accumulated to a final concentration of 3.6 ± 1.3 mM (Figure 1C) and there was an almost constant release of ^13^C_DIC_ from the degradation of protein across the experiment (Figure 1G).

In PAOM incubations, a H_2_ peak appeared at day 7 (17 ± 13 μM, Figure 1D), which decreased by day 28. Methane accumulated from day 7 to approximately day 35 (9.7 ± 7.6 μM). Sulfide concentrations showed a large variability, and unlike the other incubations showed an increasing trend throughout the incubation period and reached a maximum of 0.80 ± 0.64 mM (Supplementary Figure S3). The label incorporation in DIC slowed down after day 35 and lower levels were reached compared to the protein and acetate incubations (Figure 1H). The concentrations of metabolites (H_2_ and CH_4_) differed between incubations with labeled and unlabeled PAOM (Supplementary Figure S4). However, the same phases of hydrogen production and consumption as well as methane production were discernible with labeled and unlabeled PAOM.

### Total Community Composition by 16S rRNA Gene Amplicon Sequencing

**Figure 2:**
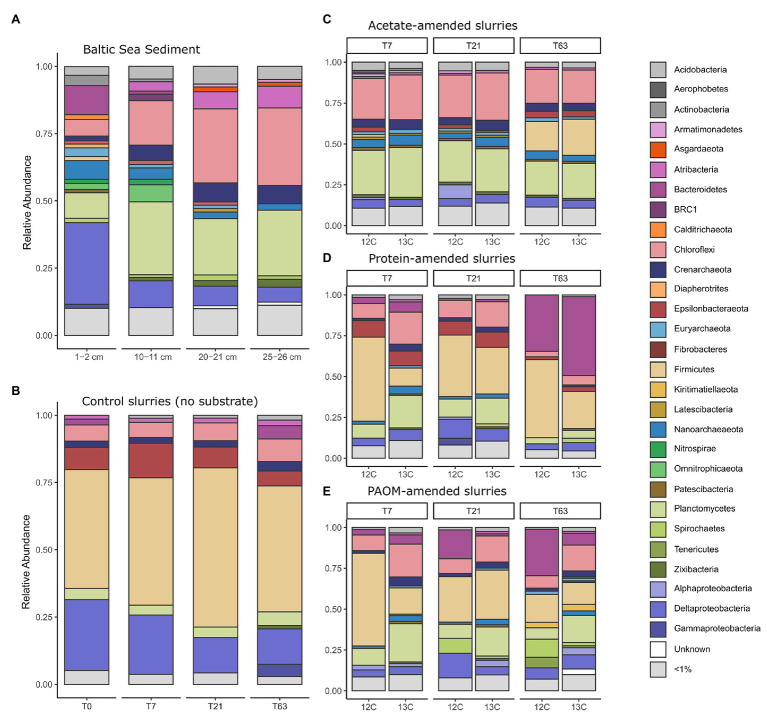
Taxonomic composition of the 16S rRNA gene diversity across depth in the original sediment samples **(A)** and across time in constructed sediment slurries **(B–E)** with **(B)** the unamended sediment slurries, the sediment slurries amended with **(C)** acetate, **(D)** protein and, **(E)** particulate algal organic matter (PAOM). The total microbial community composition is shown for both unlabeled (marked ^12^C) and labeled substrate (marked ^13^C) slurries for **(C–E)** and by combining all the DNA stable isotope probing (DNA-SIP) fractions. Colors correspond to Class level taxonomy for Proteobacteria and phylum level taxonomy for all other groups.

Microbial community analysis by 16S rRNA gene amplicon sequencing of the surface (1–2 cm) and deeper sediments (horizons at 10–11, 20–21, and 25–26 cm) revealed 2,227 OTUs in total, with 545 ± 118 OTUs by sediment horizon (Figure 2A). The largest proportion of analyzed sequences in the surface sediment corresponded to the Deltaproteobacteria class (30.4%), Bacteroidetes (10.8%), Planctomycetes (9.5%), Nanoarchaeota (7%), and Chloroflexi (6.1%). In the deeper sediment at 25–26 cm the relative abundance of Chloroflexi (28.8%), Planctomycetes (24.3%), Atribacteria (8.2%), and Crenarcheaota (6.8%) increased substantially compared to the surface sediment.

Sediments were stored intact at 4°C in the dark for 18 months before they were used as inoculum for the sediment slurries. Due to the long storage period, we consider the inoculum not as a direct representative of the *in situ* conditions, but as a source of organisms for enrichments, that are defined by provided organic matter type. A preincubation of 2 weeks was used in slurries before substrate addition to allow for the degradation of any OM made available by the mixing of the sediment matrix. As a consequence, the microbial community at the start of substrate additions (i.e., control at time = 0, Figure 2B) differed considerably from the total original sediment community and was comprised mainly of taxa affiliated to the phylum Firmicutes (44.2%), the Deltaproteobacteria class (26.3%), and the phyla Epsilonbacteraeota (8.3%), Chloroflexi (6.0%), and Planctomycetes (4.2%), with minor contributions of the Crenarchaeota (2.3%), Bacteroidetes (2.2%), and Atribacteria (1.5%; Figure 2B).

In unamended control incubations, the microbial communities did not change substantially over time as compared to the initial slurry composition with a slight increase in the relative abundance of sequences corresponding to Firmicutes by day 21 (59.2%) and an increase of Bacteroidetes (4.9%), Gammaproteobacteria (4.6%), and Zixibacteria (1.2%) after 63 days of incubation. In sharp contrast, the microbial community composition rapidly altered after substrate amendment. In acetate-amended incubations after 7 days, the relative abundance of the Firmicutes population decreased, while there was an increase in the relative abundance of members of the phyla Chloroflexi (26.2%) and Planctomycetes (28.8%) as well as Nanoarchaeota (5.6%; Figure 2C). After 63 days, the relative abundance of OTUs affiliated to the Firmicutes increased again, reaching 20.1% (Figure 2C). In protein-amended incubations, members of the Chloroflexi (13.8%) and Planctomycetes (13.8%) also increased at sampling days 7 and 21 in comparison to unamended sediment slurries, while the relative abundance of Bacteroidetes sequences increased substantially, i.e., from 5% at day 7 to 41.4% at day 63 (Figure 2D). In PAOM-amended incubations, the main changes were a similar increase in the relative abundance of members of the phylum Planctomycetes (16.7%) and Chloroflexi (14.8%) at day 7 and Bacteroidetes throughout all the sampling timepoints (4.5, 9.4, and 17.8% at day 7, 21, and 63, respectively; Figure 2E).

### Microbial Community Members Actively Degrading the Substrates as Determined by DNA-SIP

To analyze which community members incorporated ^13^C labeled and/or ^15^N labeled OM during growth from the substrate amendments, DNA was fractionated into 12 fractions ranging in density from 1.674 to 1.737 g/ml in a density gradient upon ultracentrifugation. The microbial diversity of each fraction was analyzed by amplicon sequencing of the 16S rRNA gene. The distribution of the OTUs across the fractions was used to detect the labeled OTU sequences and, therefore, the microbial community actively involved in substrate degradation (Supplementary Figures S5, S6). We used the HRSIP method (Pepe-Ranney et al., 2016; Youngblut et al., 2018b), where OTUs that were more abundant in the heavy fractions of labeled incubations compared to the heavy fractions of unlabeled incubations are categorized as labeled (see experimental procedures for the method of calculation).

**Figure 3:**
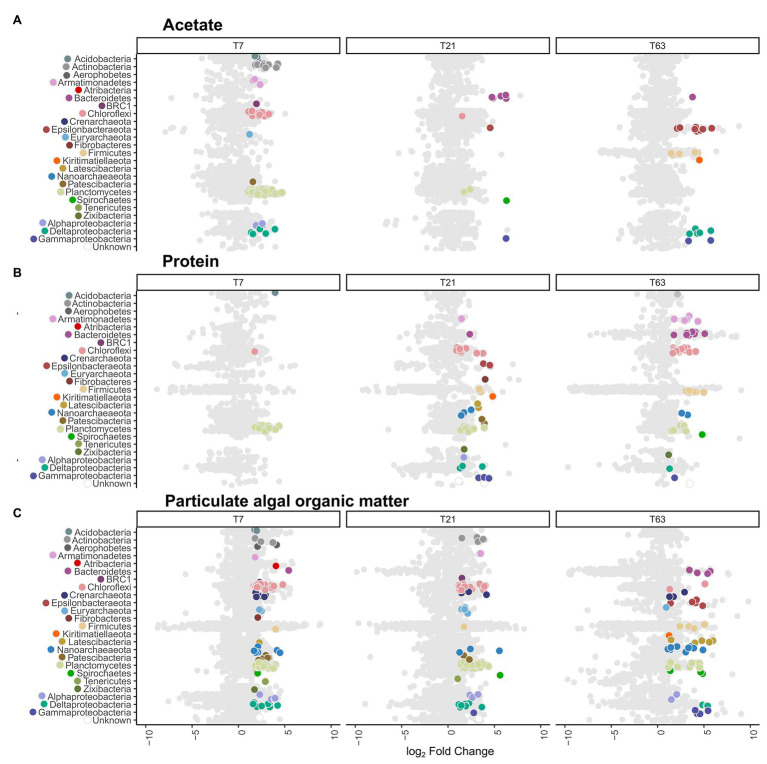
Distribution of active operational taxonomic units (OTUs) and their taxonomy compared to all OTUs. Data shown is ordered by substrate type and sampling timepoints day 7 (T7), day 21 (T21), and day 63 (T63). The x-axis depicts the difference in abundance between the heavy fractions of the labeled and unlabeled incubations as the log2 fold change calculated with DeSeq2 in the high-resolution DNA-SIP (HRSIP) workflow as indicated in the Experimental procedures. **(A)** Acetate-amended incubations, **(B)** Protein-amended incubations, and **(C)** PAOM-amended incubations. Each dot represents an individual OTU. Light gray dots represent unlabeled OTUs, while colored dots are OTUs were determined as labeled by the HRSIP analysis. Dots are color-coded by taxonomic information with colors corresponding to the ones used in Figure 2.

By application of this HRSIP method, we identified a total of 410 labeled OTUs across the nine different treatments (Figure 3; Supplementary Table S1). In the acetate-amended incubation experiments, a total of 94, 14, and 24 labeled OTUs at day 7, 21, and 63, respectively were determined. These OTUs made up a total of 13.7, 0.6, and 14.8% of the total 16S rRNA gene reads (summed across all fractions) from the samples at day 7, 21, and 63, respectively. Except for one OTU at day 7, which was related to the Euryarchaeota, all other sequences were bacterial (Figure 3A). At day 7 in the acetate-amended incubations, the majority of labeled OTUs were affiliated with the phylum Planctomycetes (43 OTUs, 9% of total reads in labeled incubation), with the next most abundant phylum being Chloroflexi (11 OTUs, 2.2%). The third most abundant phylum labeled in acetate consumption was Actinobacteria (10 OTUs, 0.5%). At day 21, few OTUs were labeled with the most of them affiliated to the phylum Bacteroidetes (five OTUs, 0.1%). At day 63, the most OTUs that were labeled were related to the Epsilonbacteraeota (eight OTUs, 0.2%) and Deltaproteobacteria class (Proteobacteria phylum; five OTUs, 0.1%). The labeled Epsilonbacteraeota were further classified into Sulfurovum, (three OTUs), Arcobacter (two OTUs), Sulfurimonas (two OTUs), and Sulfurospirillum (one OTU) at the genus level (Supplementary Table S4). The Firmicutes phylum also had several labeled OTUs (six OTUs) affiliated to the order Clostridiales, and one of them was alone responsible for about 14% of the total number of 16S rRNA gene reads detected in the incubation. Three of these OTUs were classified as Desulfotomaculum, the three others (including the abundant OTU) were unclassified at the class level.

In the slurries amended with protein, the number of labeled OTUs was 14, 55, and 53 at day 7, 21, and 63, respectively (Figure 3B). These OTUs amounted to a total of 3.5, 4.9, and 2.5% of total reads in labeled incubations at day 7, 21, and 63, respectively. At day 7, almost all labeled OTUs were affiliated with the phylum Planctomycetes (11 OTUs, representing 2.0% of total reads in incubations with labeled substrate). At day 21, in addition to Planctomycetes (10 OTUs, 1.1%) the labeled OTUs belonged to the Chloroflexi (11 OTUs, 1.9%). In addition, seven OTUs (0.6%) were labeled belonging to the Proteobacteria, three OTUs (0.4%) from the class Deltaproteobacteria and 3 (0.04%) from the class Gammaproteobacteria. At day 63, the labeled OTUs had a similar taxonomic composition as at day 21, with Chloroflexi (15 OTUs, 1%) as the most abundant phylum, and Planctomycetes as the second most abundant phylum (nine OTUs, 0.6%). In addition, OTUs from the phylum Bacteroidetes (eight OTUs, 0.3%) and Firmicutes (seven OTUs, 0.1%) were abundant. From the archaeal domain, at day 21, six OTUs (0.6%) were defined as labeled, three OTUs (0.3%) from the phylum Nanoarchaeota and two OTUs (0.06%) from the phylum Diapherotrites. At day 63, labeled OTUs were detected only in the Nanoarchaeota phylum (two OTUs, 0.02%).

In the incubation experiments with PAOM the highest number of labeled OTUs was detected with 124, 116, and 71 labeled OTUs at day 7, 21, and 63, respectively (Figure 3C). These labeled OTUs represented 23.5, 16.9, and 3.8% of total reads, respectively. As in the other substrate incubations, at day 7 the Planctomycetes (38 OTUs, 7.3%) were the most abundant in the labeled community, followed by the Chloroflexi (30 OTUs, 6.9%). In addition, 13 OTUs (1.8%) from the Proteobacteria became labeled, mainly from the class Deltaproteobacteria (seven OTUs, 1.5%). In total archaeal, 18 OTUs (3.6%) were labeled at day 7, belonging mainly to the Crenarchaeota (seven OTUs, 1.3%) and Nanoarchaeota (seven OTUs, 1.4%). At day 21, the labeled microbial community was similar to that at day 7 with Planctomycetes (52 OTUs, 7.8%) dominating, followed by Chloroflexi (21 OTUs, 3%) and Proteobacteria (11 OTUs, 1.3%), with seven (1.1%) of them belonging to Deltaproteobacteria. For the Archaea (10 OTUs, 2.4%), the Euryarchaeota had the most labeled OTUs (four OTUs, 0.6%). At day 63, label incorporation in Planctomycetes (11 OTUs, 0.8%) and Chloroflexi (two OTUs, 0.1%) had decreased, while that in the Proteobacteria (11 OTUs, 0.4%) stayed at a similar level. The Latescibacteria (five OTUs, 0.2%), Epsilonbacteraeota (five OTUs, 0.3%), and Bacteroidetes (four OTUs, 0.06%) as well as Firmicutes (four OTUs, 0.06%) had more labeled OTUs. A total of 15 archaeal OTUs (1.2%) were labeled with Nanoarchaeota Woesearchaeota (nine OTUs, 0.7%) as the most common class.

**Figure 4:**
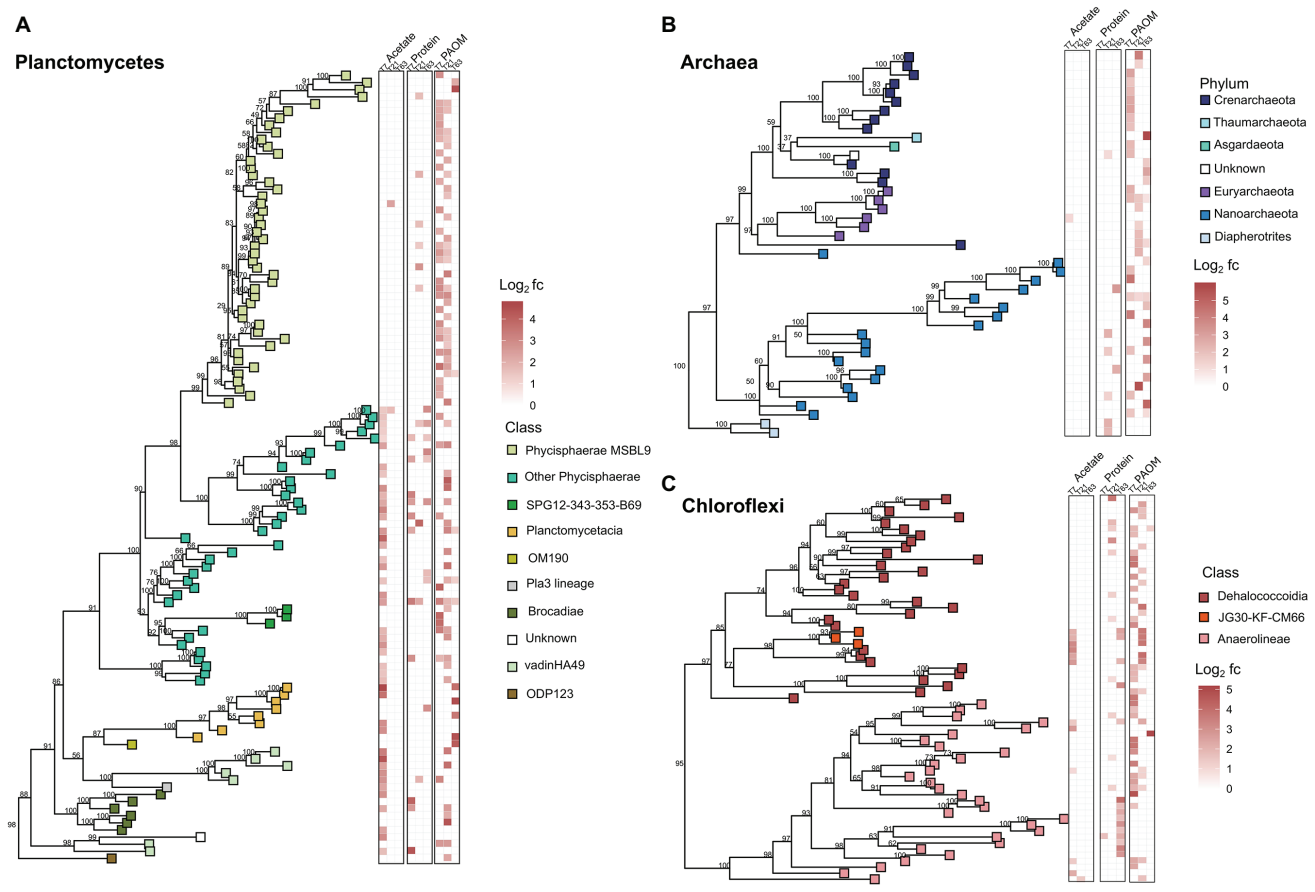
Phylogenetic trees showing the taxonomic relationship of the 16S rRNA gene fragments of the OTUs of the taxonomic groups with most labeled OTUs in the DNA-SIP analysis of organic matter (OM) degradation. **(A)** labeled OTUs of Planctomycetes, **(B)** labeled OTUs of Archaea, and **(C)** labeled OTUs of Chloroflexi. Heatmaps show the log2 fold change calculated by HRSIP compared across the different treatments and sampling timepoints. Colors correspond to class level or phylum level taxonomic group as defined by sequence analysis. Values shown in tree are bootstrap support values.

As the phyla Planctomycetes, Chloroflexi, and the domain Archaea were the most abundant throughout the labeled communities at all timepoints and substrates, we analyzed the phylogenetic relationships of these OTUs in more detail to detect community differences arising from the treatments (Figure 4). The phylogenetic analysis showed clustering of the labeled groups in different substrate incubations at a deeper taxonomic level. In the Planctomycetes, the class Phycisphaerae was divided further at order level based on substrate affinity. The order MSBL9 was most labeled during initial stages of PAOM degradation, while the uncultured orders CCM11a, mle1–8, and Phycisphaerales were active also in protein‐ and acetate-amended incubations in combination with other diverse classes of Planctomycetes like the Planctomycetaceae, and members of the vadinHA49 and the OM190 clusters (Figure 4A). OTUs affiliated to the archaeal domain were almost solely labeled in the assimilation of the complex OM substrates, i.e., protein and PAOM, and not in the assimilation of acetate. In protein-amended incubations, only archaeal OTUs related to the Nanoarchaeota were labeled, while in the PAOM-amended incubations also OTUs affiliated to the Crenarchaeota were detected (Figure 4B). For the phylum Chloroflexi, a subset of OTUs affiliated to Dehalococcoidia and closely related OTUs falling into the class JG30-KF-CM66 were labeled during acetate assimilation (Figure 4C). In protein-amended incubations, Dehaloccoidia OTUs were also labeled at day 21 but at day 63 the labeled Chloroflexi were predominantly affiliated to the class Anaerolineae. These latter OTUs differed, however, from the Chloroflexi Anaerolineae OTUs that were active in the incubations with PAOM (Figure 4C).

## Discussion

The objective of this study was to identify the prokaryotic groups originating from anoxic sediments and involved in the anaerobic degradation of complex algal organic matter. While the amount of substrate added in this experiment does not represent conditions *in situ*, the additions were designed to ensure labeling of the microbial community and test for theoretical niche separation based on substrate type under sulfate-depleted conditions. In addition, the utilized complex substrates are different from the OM present in the sediment; however, we consider that the molecular composition of algal biomass will represent some of the most common OM molecules processed by microorganisms in sediments. During sediment slurry incubations, different phases of anaerobic digestion were identified by the evolution of the concentration of hydrogen, acetate, and methane. These are metabolites, which can accumulate during hydrolysis of complex OM, fermentation, acetogenesis, and methanogenesis. The formation of hydrogen, acetate, and methane in protein‐ and PAOM-amended incubations was considerably higher than in unamended control incubations (~3x, Figure 1), showing that the provided complex organic substrates were primarily responsible for driving the activity of the microbial communities in the incubations. Addition of labeled acetate resulted in the formation of labeled DIC (Figure 1F), indicating active utilization of acetate. From the shifts in total community composition (Figures 2, 3) it is clear that there may be many members in the microbial community that are active, but are not involved in the anaerobic processing of OM. In addition, the provided complex substrates were only partially labeled, and therefore, the OTUs identified as labeled represent important members of the organic matter assimilating community, but do not show all organisms involved in the degradation process. Due to the how the substrate was produced, we assumed that the labeling of all different fractions of the organic matter were relatively uniform, allowing the identification of the important degraders of complex organic matter mixtures. It should also be noted, that micro-organisms secreting enzymes for the degradation of complex organic matter are possibly not the same as the microbes assimilating the degradation products. Our focus here is detecting direct evidence on activity in OM assimilation, so the microbial community incorporating labeled isotopes in their DNA is referred to as the active community in this study.

In all substrate incubations, a high proportion of the microbial community actively degrading the amended substrate was related to the phyla Planctomycetes and Chloroflexi, revealing that members of these phyla are important degraders of OM. In the following sections, we discuss the microbial communities that were potentially active in the primary fermentation of complex OM to intermediate products, possible microorganisms responsible for secondary fermentation in the acetogenic phase, and finally microorganisms active in utilizing acetate as well as other possible terminal OM degradation processes. While, we realize that taxonomy inferred from the 16S rRNA gene does not delineate functional groups of organisms, we have used this taxonomy here, to be able to compare to previous findings dealing with these microbial communities in sediments.

### The Identity of Initial Fermenters of Complex Carbon Substrates

The PAOM and protein substrate represent two fractions of complex OM pools that would be available to sediment microorganisms. Strikingly, while the sediment community shifted during storage and preincubation to a majority of *Clostridiales* (*Firmicutes*), the OM amendment resulted in activity of the main original groups present in the deeper sediment layers (Figure 2). Members of the *Firmicutes* form the known endospore communities in Baltic Sea sediments (Cupit et al., 2019). While it is not likely that we have retrieved difficult-to-extract endospore DNA, it is possible that their presence in high relative abundance indicates a life strategy adjusted to survival in conditions not favorable to other organisms. Despite this, the activation of heterotrophic taxa upon amendment with OM in incubation clearly indicates that the microbial community in the inocula still contained sedimentary organisms reliant on OM degradation. Both OM substrates were actively hydrolyzed and induced fermentative metabolisms (Figure 1), resulting in similar microbial communities based on 16S rRNA gene microbial analysis (Figures 2D,E). However, after 7 days, the PAOM degradation resulted in much more labeled OTUs and sequences (23.5% of reads) compared to protein degradation (3.5% of reads). This difference can be explained in several ways. Based on our experiments, it is not possible to compare the amount of label taken up in the DNA between the two substrate experiments, therefore, it could indicate that there was a highly specialized microbial community able to degrade proteins. However, it is also possible that the hydrolysis and fermentation products of protein were not assimilated to the DNA, but rather used as an energy source, or as building blocks for new proteins. The constant release of ^13^C-labeled DIC in protein amended incubations (Figure 1G) supports the active degradation of proteins, without their assimilation to DNA. An analysis of labeled proteins or an approach following the flow of amino acids, like BONCAT (Hatzenpichler et al., 2014) could shed further light on this.

The contrasting labeling of a high number of the microbial community in the PAOM-amended incubations coincided with a peak production of H_2_, indicating active fermentation, as expected by the absence of TEAs. In addition, we detected dominating taxonomic groups from the orders MSBL9 (phylum Planctomycetes, class Phycisphaerae) and the Anaerolineales (phylum Chloroflexi), which had at least double of the amount of active OTUs compared to all other taxa, suggesting their role as specialized initial fermenters. The composition of the large, metagenome-assembled genomes (MAGs) from Planctomycetes affiliated to MSBL9 (Phycisphaerae) in cellulose-degrading anaerobic digesters (Vanwonterghem et al., 2016) and in anoxic estuary and subsurface aquifer sediments (Baker et al., 2015; Anantharaman et al., 2016; Robbins et al., 2016) revealed extended abilities to utilize the degradation products of polysaccharides (Spring et al., 2018). Spring et al. (2018) also obtained three isolates of the MSBL9 clade from hypersaline mats and marine sediments, which were obligately anaerobic and metabolized carbohydrates to ethanol, acetate, formate, and lactate. Our data fully supports the role of the Phycisphaerae MSBL9 order as initial degraders of complex carbohydrates derived from algal material. In addition to the MSBL9, the Chloroflexi class Anaerolineales was highly active in the initial phase of the PAOM incubation experiment. Isolated strains affiliated to the Anaerolineales are characterized by obligate anaerobic growth, filamentous morphology, and the chemo-organotrophic metabolism of sugars and polysaccharides (Imachi et al., 2014), supporting our findings. Specific archaeal taxa also appeared to be specialized in the initial phase of PAOM utilization in our incubation experiments. Several labeled OTUs corresponded to the Crenarchaeota Bathyarchaeota in the early phase of the PAOM-amended but not in the protein-amended incubations (Figure 3). While Bathyarchaeota have been previously linked to protein utilization using MAGs obtained from sediments (Lloyd et al., 2013; Lazar et al., 2015) the potential to utilize carbohydrates has also been supported by the finding of genetic potential for cellulosomes in marine secretomes (Orsi et al., 2018). Like previous work that was not able to detect activity with protein amendment (Seyler et al., 2014; Yu et al., 2018), our results indicate that the Bathyarchaeota are specialized in the hydrolysis of carbohydrates. The lack of labeling of the OTUs of Bathyarchaeota, which generally host a large amount of peptidases in their genomes (Lloyd et al., 2013; Zhou et al., 2018; Steen et al., 2019), in our protein incubation could also have to do with the use of amino acids as an energy source rather than for growth as discussed previously.

### Potential Secondary Fermenters in the Anoxic Sediment Slurries

In both the PAOM‐ and protein-amended incubations the labeled communities at day 21 were diverse with no dominant groups. All H_2_ was utilized and the acetate concentration increased in protein-amended slurries, indicating active secondary fermentation of the monomers released from the primary fermentation processes. Overlapping active taxonomic groups between the two substrate incubations, a lack of dominating taxonomic groups, and an increase of the number of OTUs labeled in the protein incubations compared to the initial sampling day, also points to a process dependent on intermediate degradation products like VFAs and alcohols. For example, the Dehaloccoidia (Chloroflexi) were active, but with a small number of OTUs across several different orders with both substrates, in contrast to the high number of OTUs affiliated to one clade in PAOM incubations. This hypothesis is also supported by indications of the Dehaloccoidia as possible degraders of secondary fermentation products from the findings of the genetic potential for the degradation of fatty acids and other organic acids identified in MAGs from subsurface sediments (Hug et al., 2013; Wasmund et al., 2014) and the anoxic Black Sea water column (Suominen et al., 2019). Another active group with several OTUs in both substrate incubations was the Deltaproteobacteria class. Many members of this class are sulfate-reducing bacteria able to utilize VFAs, acetate and H_2_, which are known intermediate fermentation products (Dyksma et al., 2018; Jochum et al., 2018; Petro et al., 2019). A similar labeling trend in active Deltaproteobacteria members across several different orders with both substrates supports the notion that this is indicating degradation of intermediate products (Supplementary Table S1), and that trace amounts of sulfate were present or recycled to support sulfate-reducing metabolism.

In addition to the bacterial communities, the labeling across diverse groups of the Nanoarchaeota. Woesearchaeota in both PAOM and protein incubations could indicate a role in the degradation or assimilation of secondary fermentation products. The Woesearchaeota are a relatively poorly defined taxonomic group, mainly found in anoxic environments (Liu et al., 2018). They belong to the DPANN superphylum of Archaea, which have small genomes and probably depend on interactions with other organisms for many of their nutritional needs (Castelle et al., 2015, 2018; Dombrowski et al., 2019). They were found in labeled protein-amended incubations in the anoxic Black Sea water column (Suominen et al., 2019), which could suggest that this group is assimilating labeled carbon from amino acids also in these incubations.

### The Microbial Community Assimilating Acetate

To separate acetate-utilizing organisms from those involved in the process of the anaerobic breakdown of complex OM, the assimilation of acetate was examined in separate slurries. Acetate is a major intermediate carbon compound in anaerobic digestion and is produced in secondary fermentation of VFAs, alcohols, and amino acids (Jørgensen, 2006; Lever, 2012; Arndt et al., 2013). In Baltic Sea sediments, acetate has been measured in μM concentrations, often kept at low concentrations because of equilibrium conditions between production and consumption (Glombitza et al., 2019). The increase in the concentration of ^13^C-labeled DIC and the DNA labeling of specific prokaryotes demonstrates that acetate was consumed. However, the decrease of acetate concentrations also in the liquid phase of killed control incubations indicates that this consumption did not affect the large pool of acetate provided in the amendment (Figure 1).

Previous DNA-SIP studies into acetate assimilation have been made in sulfate-reducing Arctic surface sediments (Müller et al., 2018) and coastal sediments (Vandieken and Thamdrup, 2013; Na et al., 2015), as well as in the Baltic Sea at the water column redoxcline (Berg et al., 2013). These studies have identified major sulfate, or iron/manganese reducing taxa like Deltaproteobacteria, Arcobacter, and Colwellia as acetate metabolizers (Berg et al., 2013; Vandieken and Thamdrup, 2013; Müller et al., 2018). Only few of these known TEA-reducing orders were revealed to be active in our slurries after 7 days of incubation. In fact, the most labeled OTUs in the initial acetate amended community belonged to the phylum Planctomycetes (Figure 3). However, the taxonomy of the active organisms was distinct of those active in other substrate amendments (Figure 4). The most abundant were the Phycisphaerae, but in contrast to the PAOM incubations, they belonged to the orders Phycisphaerales and CCM11a. Cultured Phycisphaerales have been isolated from algal cells (Fukunaga et al., 2009; Yoon et al., 2014), and CCM11a has been found abundant in clone libraries from iron hydroxide deposits (Storesund and Øvreås, 2013). Interestingly, we also detected labeling in other Planctomycetes classes like the vadinHA49. This class has been previously detected as heterotrophic with low organic carbon uptake in slurries made from surface sediments of freshwater ponds (Coskun et al., 2018). Our finding of acetate assimilation could explain the mechanism by which these organisms became marginally labeled in incubations with high molecular weight OM (Coskun et al., 2018). Similarly, inside the group of active Chloroflexi there was a specialized cluster of acetate-assimilating OTUs (Figure 4C), affiliated with Dehalococcoidia as well as the less abundant class JG30-KF-CM66 (proposed name Ca. Bathosphaeria; Mehrshad et al., 2018). In our study, these groups were also found to be active in the later stages of complex carbon degradation, supporting their role linked to acetate assimilation. The comparison of acetate incubations to complex carbon (i.e., PAOM and protein) amended slurries shows how the microbial community is highly divided into the separate functional groups, which was also reported in a previous study testing sequential degradation of OM (Müller et al., 2018).

Contrastingly, at the later stage of the acetate incubations (i.e., day 63) a diverse community related to organisms with known TEA respiration capabilities was identified, while ^13^C-labeled DIC concentrations also point to active oxidation of acetate. The later onset of TEA respiration could have to do with a lag phase caused by the extended storage period, or presumably low concentrations of available TEAs in the slurries. The most abundant taxa was affiliated to an unknown family of the order Clostridiales (Firmicutes), but other labeled OTUs from the Firmicutes were classified to the genus Desulfotomaculales, a known sulfate-reducing family (Aüllo et al., 2013). The sequences were most closely related to the genus Desulfofarcimen, which has been identified in connection with acetate amendment previously (Widdel and Pfennig, 1977; Watanabe et al., 2018). In addition, diverse Epsilonbacteraeota Campylobacterales affiliated to the genera Sulfurovum, Sulfurospirillum, Arcobacter, and Sulfurimonas incorporated labeled acetate. These taxa are known for various sulfur metabolisms, like the reduction of thiosulfate and elemental sulfur in connection with the oxidation of small organic compounds (Campbell et al., 2006; Han and Perner, 2015; Waite et al., 2017), or the use of acetate as a carbon source in otherwise autotrophic sulfur oxidizing metabolism (Gevertz et al., 2000). However, a major part of Epsilonbacteraeota are also connected to autotrophic metabolisms enabled by sulfur-oxidation in sulfidic environments (Grote et al., 2008; Glaubitz et al., 2014; Waite et al., 2017), making it possible that at this stage labeling occurred by the autotrophic uptake of ^13^C-labeled DIC formed from labeled acetate. Taken together these results indicate a slow initiation of acetate oxidation, and the recycling of diverse sulfur species.

### Terminal OM Degradation Processes and the Identity of Labeled Microorganisms

One of the most drastic contrasts seen when comparing the different substrate incubations was the differences in terminal stages of the degradation process. The degradation of PAOM resulted in an activation of methanogenic processes, while protein degradation ended with the accumulation of acetate and both protein‐ and acetate-amended incubations continued with an accumulation of ^13^C-labeled DIC until the end of the incubations (Figure 1). In addition, in PAOM incubations, an increase in sulfide concentrations (~1 mM increase) compared to the protein‐ and acetate-amended incubations was evident (Supplementary Figure S3), indicating that the difference in substrate induced distinct metabolisms in both secondary and terminal stages in the OM degradation.

In methanogenic conditions, acetate can also be cleaved by acetoclastic methanogens or fermented by bacteria in syntrophy with hydrogenotrophic methanogens (Zinder, 1994; Conrad, 1999; Parkes et al., 2007; Beulig et al., 2017). Previous DNA-SIP studies of acetate-assimilating microbial communities in methanogenic sediments of lakes (Schwarz et al., 2007) and rice field soils (Hori et al., 2007) have shown assimilation of acetate in a large amount by methanogenic archaea, with minor contributions from bacterial taxa. These studies were run for only 12–18 days, albeit at higher temperatures than our incubations. The lack of methanogenesis in our acetate-amended incubations and the accumulation of acetate in the protein-amended incubations is, therefore, possibly due to a slower metabolism of acetate, or a delay in the onset of syntrophic interactions between acetate-oxidizing bacteria and methanogenic archaea, suggested previously as a reason for acetate accumulation at the onset of methanogenic conditions in Baltic Sea sediments (Beulig et al., 2017).

In contrast, in the PAOM incubations a labeled archaeal community was obtained during the active methanogenic phase (days 14–28), showing label incorporation in the DNA of known methanogenic taxa. At day 21, the majority of archaeal OTUs were affiliated to the Euryarchaeota classes Methanomicrobia and Marine Benthic Group D (MBG-D; new name Thermoprofundales; Zhou et al., 2019). Methanomicrobia classified to the order Methanocellales are likely the main methane producer (Sakai et al., 2008) in our incubations. On the other hand, environmental MAGs from the order Thermoprofundales have indicated that this group is possibly involved with the degradation of peptides (Zhou et al., 2019). Their labeling in our incubations indicates either more diverse metabolisms in relation to this taxonomic group, recycling of trace amounts of peptides in the substrate, or that peptides were being recycled from inside the PAOM assimilating community.

## Conclusion

In this study, we investigated the microbial communities potentially responsible for the degradation of complex algal OM sources originating from marine anoxic sediments. Our study showed that microorganisms from OM-rich sediments such as those in the Baltic Sea can grow on complex OM pools. The high relative abundance of the taxa phylogenetically related to the labeled OTUs in the original sediment community, and especially their induction after an extended storage period of the sediments, indicates that the processing of complex OM is one of the main functions of the sediment microbial community. In addition, we detected major differences in the processes and terminal stages of OM degradation dependent on substrate composition. This shows that the high level of interactions needed for the full degradation of OM are sensitive to disturbances and are possibly induced already at earlier stages of the degradation process.

Our study shows that members of the phyla Chloroflexi and Planctomycetes are the most important degraders of complex organic matter originating from algae. The possible significance of these organisms in Baltic Sea sediments is supported by the fact that microorganisms affiliated with these phyla made up the majority (>50%) of sequences analyzed in the original sediment (Figure 2), but more research is needed in natural conditions to confirm this. In addition, we observed that specialization to the degradation and assimilation of different OM type happens at the level of taxonomic orders or at lower taxonomic levels. We were able to induce and confirm the metabolic activity of common heterotrophic microbial taxa, and show that niche differentiation to degrade specific compound types is a major factor defining the microbial community in these conditions.

## Data Availability Statement

The sequencing data presented in this study is deposited in the NCBI sequence read archive (SRA) under the bioproject number PRJNA702135.

## Author Contributions

SS did the sampling, planning, experiments, analyses, and writing. DV and IS-A helped in sample analyses and commented on the manuscript. MM helped in sampling and commented on the manuscript. JS commented on the manuscript. LV supervised the project. All authors contributed to the article and approved the submitted version.

### Conflict of Interest

The authors declare that the research was conducted in the absence of any commercial or financial relationships that could be construed as a potential conflict of interest.
