# Supplementary material for: Organic Matter Type Defines the Composition of Active Microbial Communities Originating From Anoxic Baltic Sea Sediments
Source: Front Microbiol. 2021 May 5;12:628301. doi: 10.3389/fmicb.2021.628301 (PMC8131844; doi:10.3389/fmicb.2021.628301)
Supplement: Supplementary file 1 [file Data_Sheet_1.PDF]

Supplementary Material

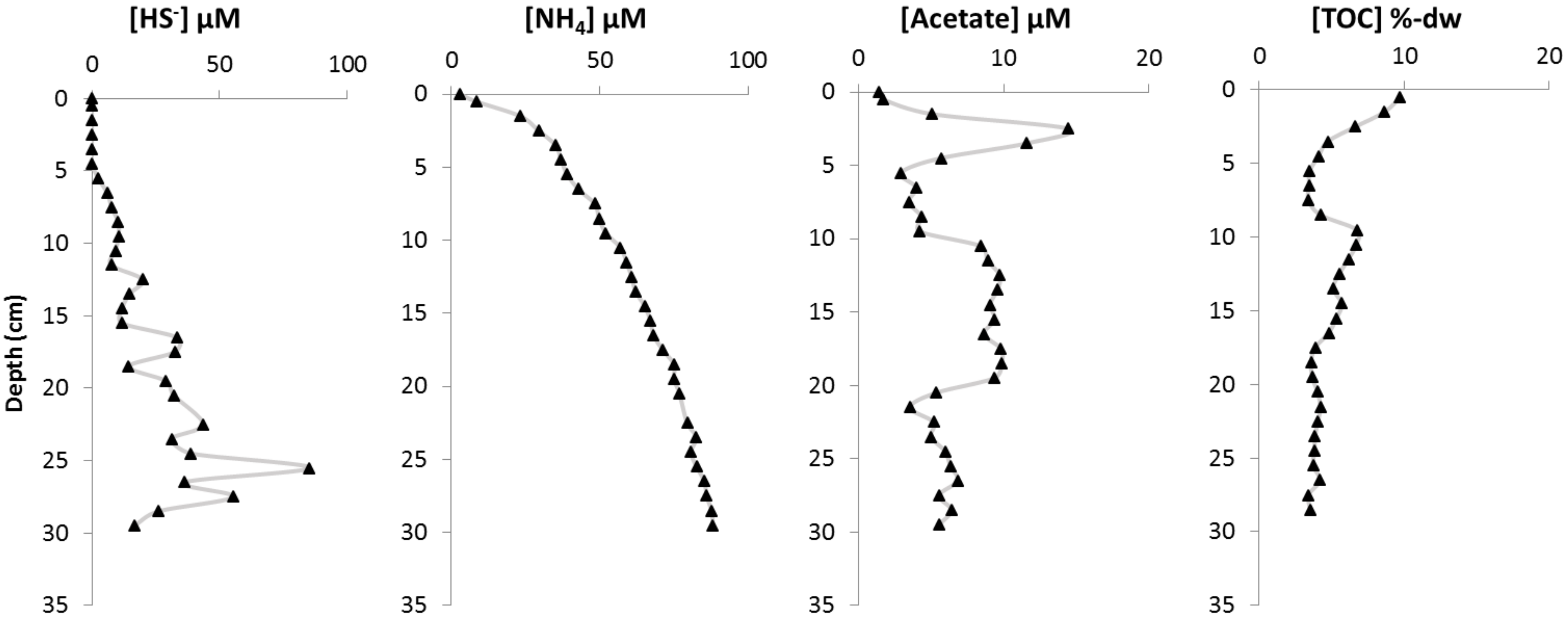

Figure S1, Concentrations of porewater chemical parameters measured from sampling site at the Baltic Sea Gotland Basin and indicative of organic matter degradation processes. TOC: total organic carbon, as %-dw: dry weight.

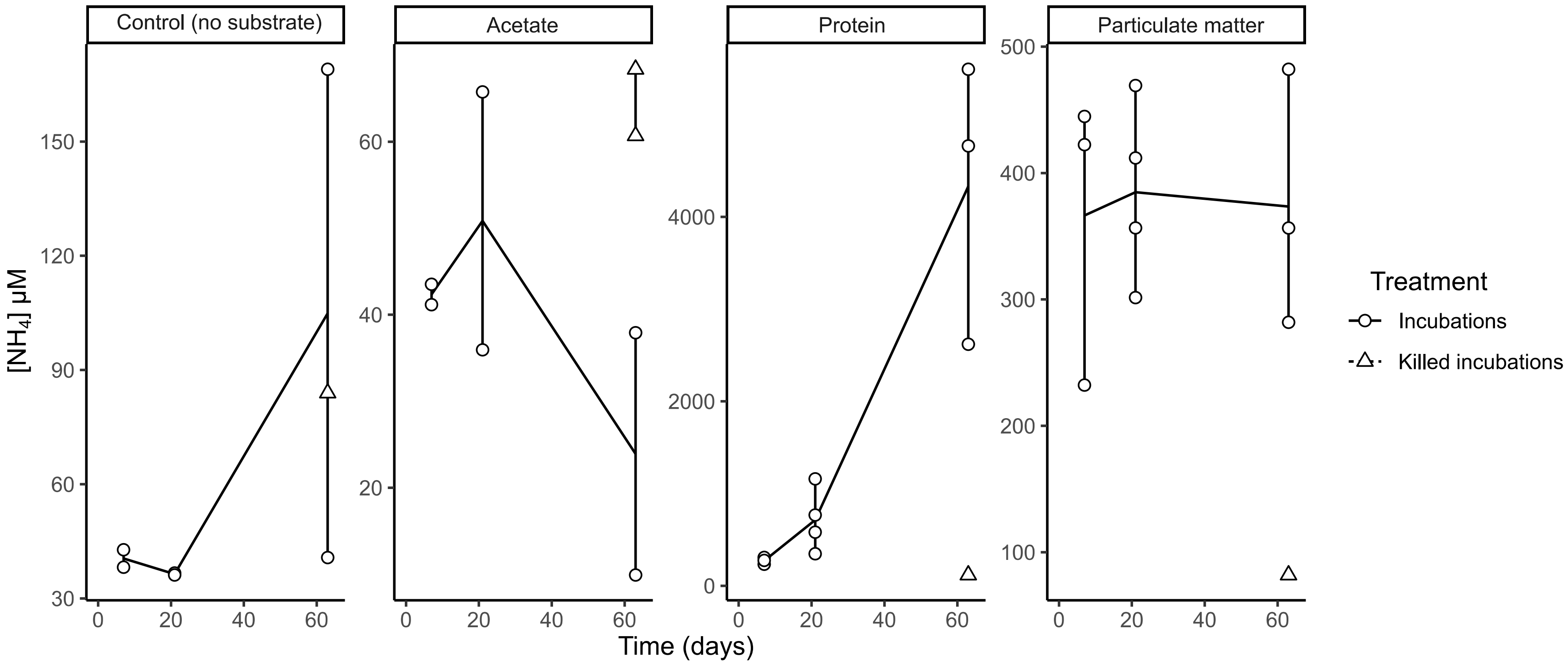

Figure S2, Ammonium concentrations measured from sediment slurries amended with different organic substrates and across sampling timepoints. Circles represent measurement from individual live incubations while triangles show the endpoint measurements from killed incubations. Note the differences in the scale of the y-axis. Measurements of replicate incubations are connected with vertical lines, and the line across timepoints runs through calculated average values of the replicate live incubations.

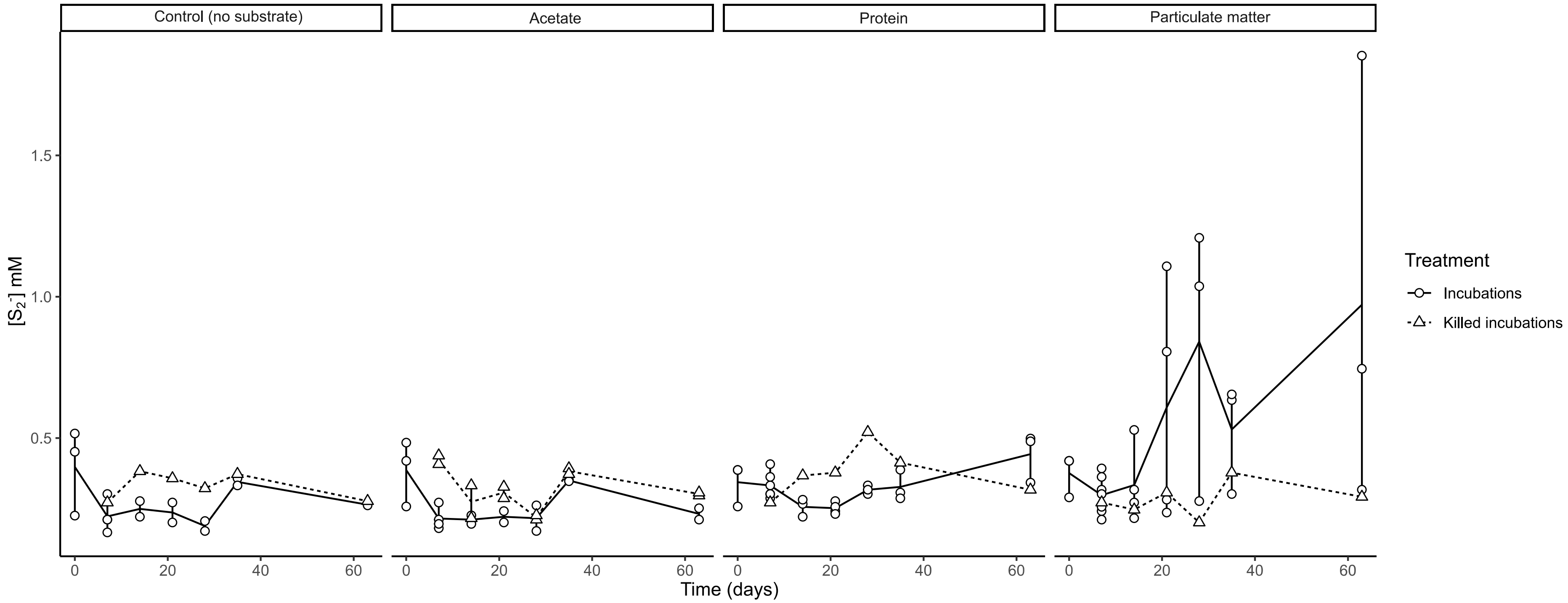

Figure S3, Sulfide concentrations measured from sediment slurries amended with different organic substrates and across sampling timepoints. Circles represent measurement from individual live incubations while triangles show the measurements from killed incubations. Measurements of replicate incubations are connected with vertical lines, and the lines across timepoints runs through calculated average values of the replicate incubations, with the solid line depicting live incubations and the dashed line killed incubations.

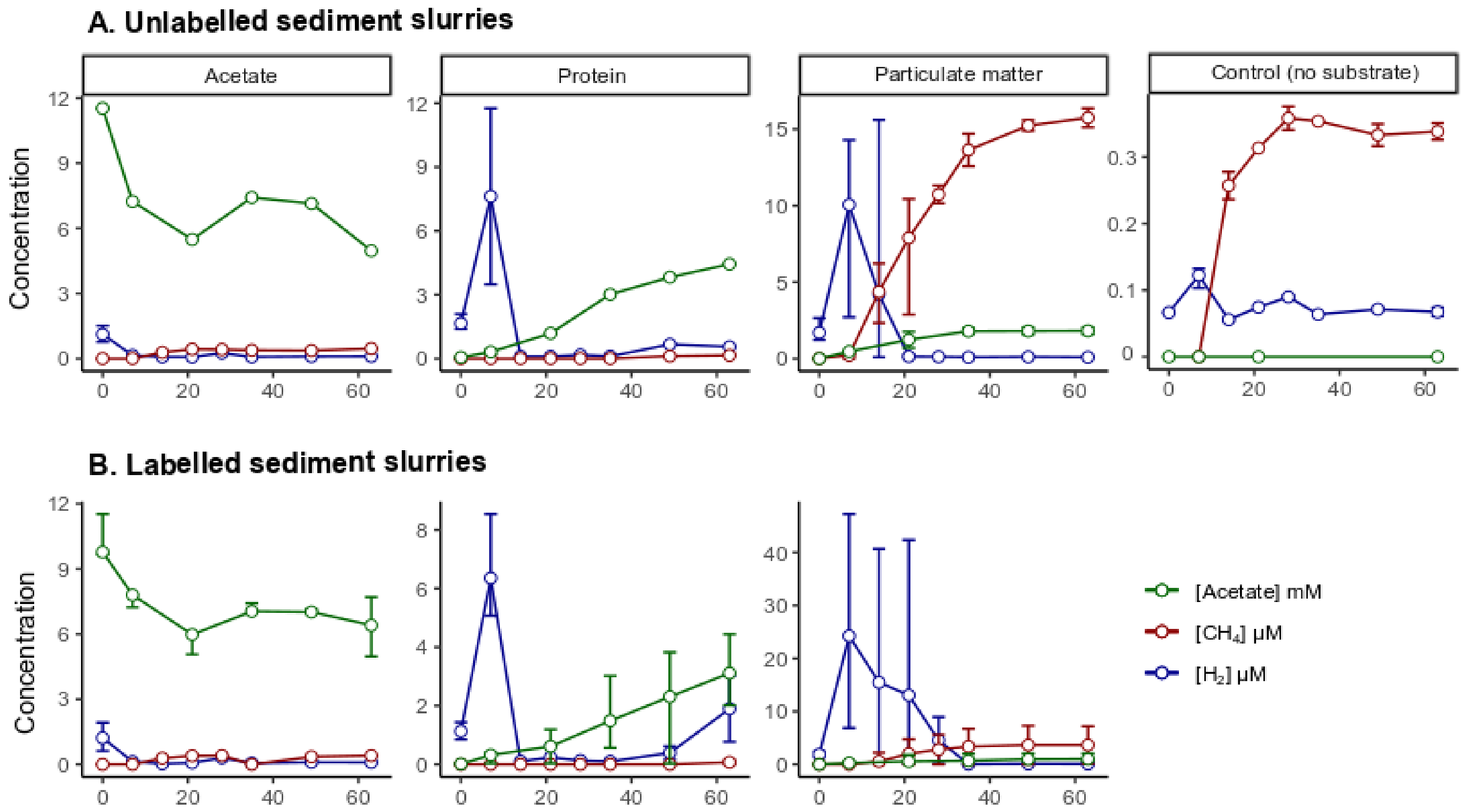

Figure S4, Measured concentrations of hydrogen (blue), methane (red) and acetate (green) in sediment slurries amended with different organic matter types A. In slurries amended with unlabelled organic substrates or no substrate (control) and B. In slurries amended with labelled organic substrates. Note the different ranges of the y-axis. Error bars depict the range of (n=2-3) values

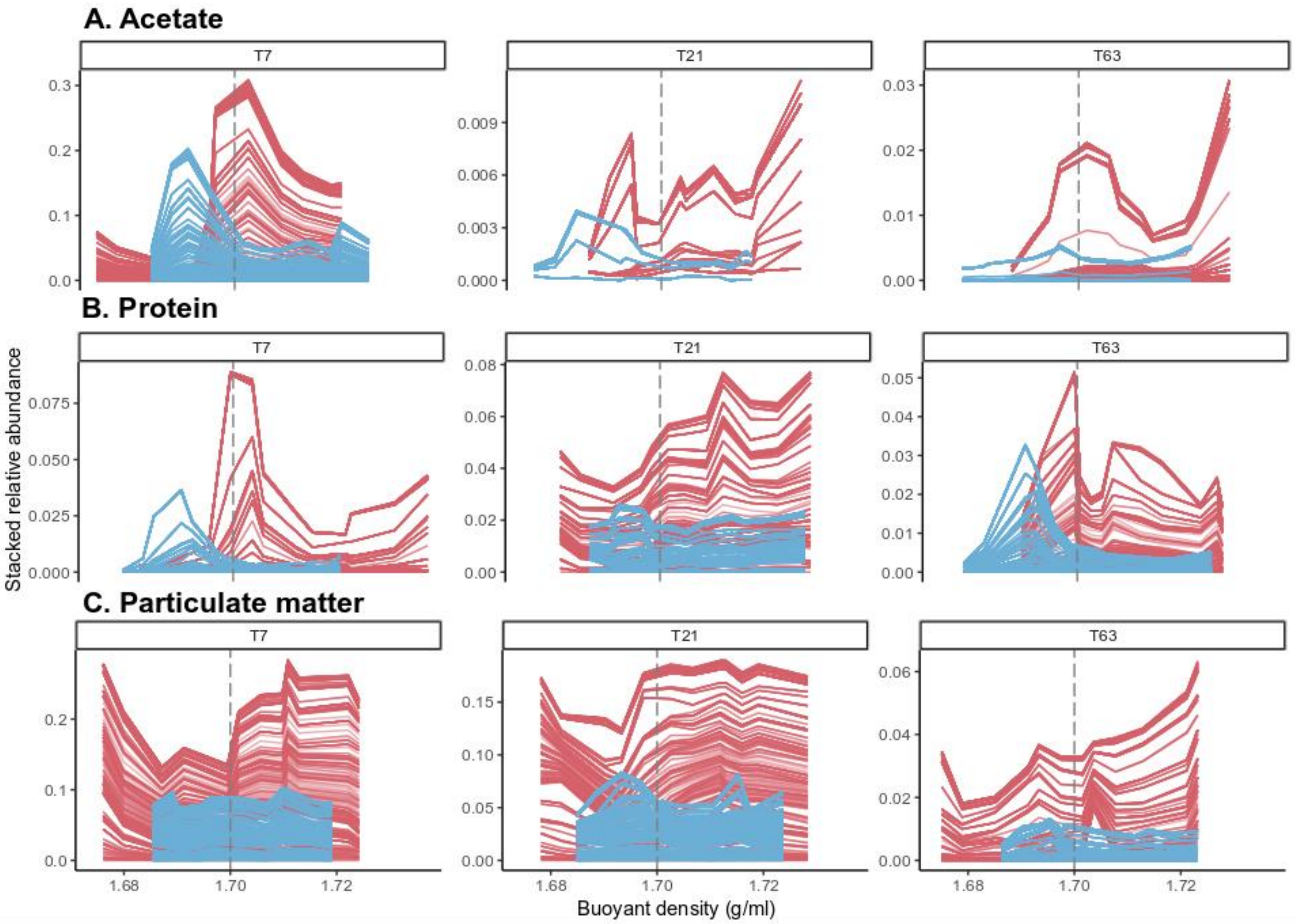

Figure S5, Distribution of labelled OTUs across density gradients by substrate type and sampling time. A. Acetate incubations, B. Protein amended incubations, C. Particulate matter amended incubations. Each tile compares the labelled (red) and unlabelled (blue) slurry incubations across one treatment. The Y-axis shows the relative abundance of the OTUs at each density stacked. Dashed lines represent the division of the density gradient to light and heavy fractions

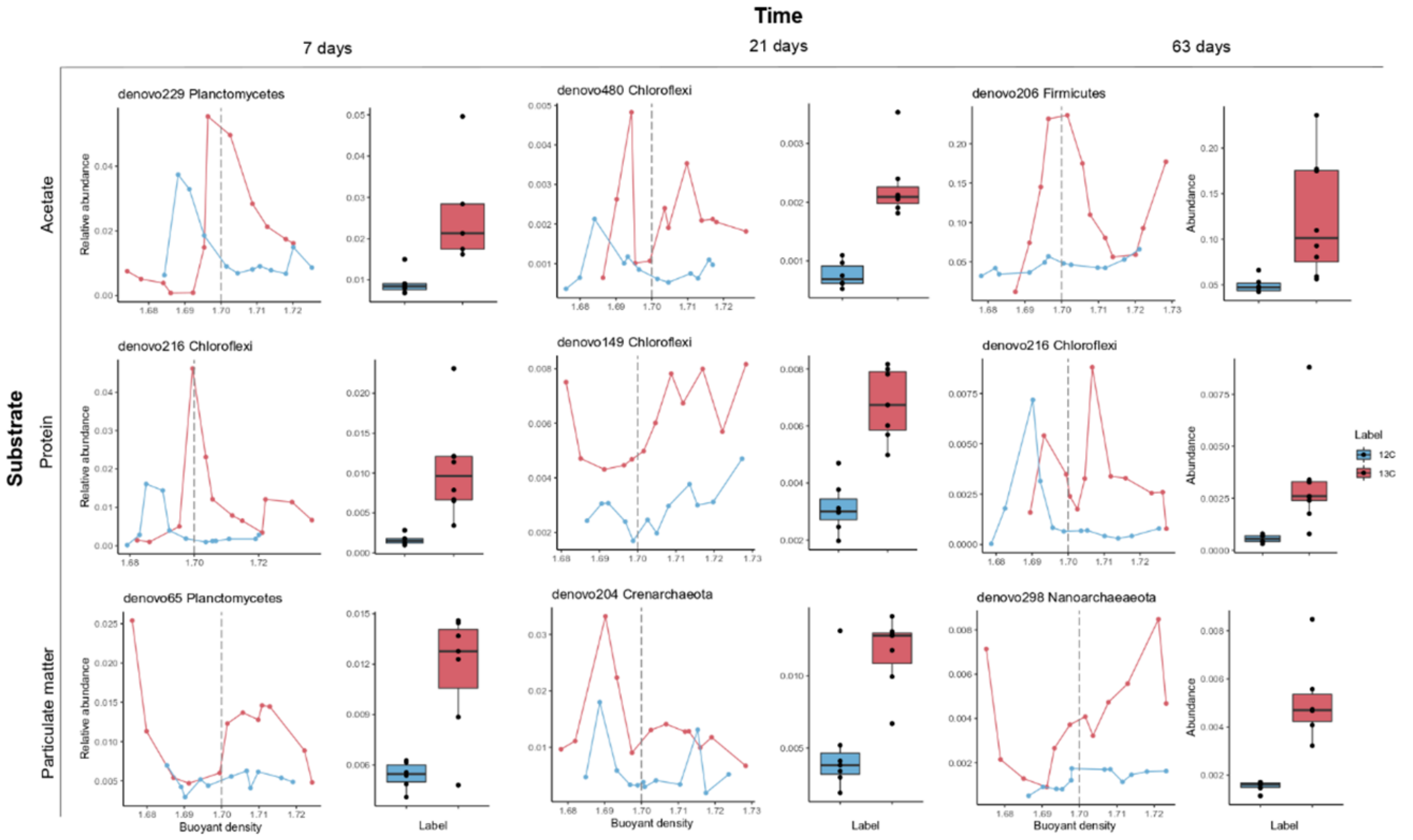

Figure S6, The distribution of the most abundant active OTU of each treatment. Shown across the gradient in unlabelled (blue) and labelled (red) incubations. The dashed line shows the limit between light and heavy fractions (1.70 g/ml). The boxplots show the relative abundance in heavy fractions compared between labelled and unlabelled incubations. Based on this abundance difference the OTUs were determined active in the HRSSIP analysis.

Table S1,Relative abundances of common orders (>1%) across the sediments, unamended slurries and active communities.

												Active groups (Relative abundance of reads in labelled incubations)								
				Sediment (cm)				Unamended				Acetate			Protein			Particulate OM		
	Phylum	Class	Order	1-2	10-11	21-22	25-26	T0	T63	T7	T21	T7	T21	T63	T7	T21	T63	T7	T21	T63
Archaea	Crenarchaeota	Bathyarchaeia		0.02	0.06	0.07	0.07	0.02	0.03	0.02	0.02							0.0131	0.0147	0.0018
	Euryarchaeota	Methanomicrobia	Methanocellales																0.0004	
		Thermoplasmata		0.02								0.0012						0.0046	0.0059	0.0037
	Nanoarchaeaeota	Woesearchaeia		0.07	0.04	0.02	0.02									0.0033	0.0002	0.0136	0.0025	0.0068
Bacteria	Acidobacteria			0.03	0.04	0.07	0.05		0.02	0.01	0.01	0.0013			0.0002		0.0004	0.0074		
	Actinobacteria			0.04								0.0051						0.0038	0.0036	
	Armatimonadetes					0.01						0.0061				0.0015	0.0012	0.0010	0.0003	
	Atribacteria	JS1			0.04	0.06	0.08	0.01	0.02	0.01	0.02	0.0002	0.0002	0.0002	0.0002	0.0002	0.0002	0.0002		
	Bacteroidetes			0.07	0.01			0.02	0.05				0.0011	0.0002		0.0002	0.0034	0.0004		0.0006
	Calditrichaeota	Calditrichia	Calditrichales	0.02																0.0006
	Chlamydiae	Chlamydiae	Chlamydiales																	0.0004
	Chloroflexi	Anaerolineae		0.04	0.05	0.12	0.15	0.04	0.06	0.04	0.05	0.0097	0.0021		0.0130	0.0082	0.0089	0.0240	0.0104	0.0002
		Dehalococcoidia		0.02	0.10	0.13	0.12	0.02	0.02	0.02	0.02	0.0025				0.0111	0.0005	0.0449	0.0121	0.0010
	Dependentiae	Babeliae	Babeliales																0.0001	0.0001
	Epsilonbacteraeota	Campylobacteria	Campylobacterales					0.08	0.06	0.13	0.08		0.0003	0.0020		0.0006				0.0031
	Firmicutes			0.01				0.44	0.47	0.47	0.59			0.1440		0.0002	0.0013	0.0002	0.0057	0.0006
	GN01														0.0009	0.0009	0.0006			
	Hydrogenedentes	Hydrogenedentia	Hydrogenedentiales														8.108E-05			
	Kiritimatiellaeota	Kiritimatiellae	Kiritimatiellales											0.0001		0.0002				0.0033
	Latescibacteria					0.01										0.0003		0.0015		0.0017
	Marinimicrobia																			0.0001
	Nitrospirae			0.01	0.02							0.0011				0.0005		0.0045	0.0048	0.0001
	Omnitrophicaeota			0.02																
	Planctomycetes	Phycisphaerae	CCM11a		0.02	0.01	0.01					0.0279	0.0012		0.0105	0.0008	0.0036	0.0013	0.0016	
			DG-20		0.03	0.03	0.02					0.0285			0.0020			0.0013	0.0116	
			mle1-8		0.02							0.0088			0.0046	0.0003	0.0004	0.0010	0.0043	0.0016
			MSBL9	0.04	0.15	0.12	0.15	0.03	0.03	0.03	0.03		0.0005			0.0078		0.0651	0.0509	0.0016
			Phycisphaerales		0.01	0.01	0.02					0.0138			0.0022	0.0012	0.0013	0.0019	0.0055	0.0043
			Pla1 lineage									0.0022							0.0011	
		Other		0.02	0.03	0.02	0.02					0.0076			0.0003	0.0008	0.0001	0.0019	0.0018	0.0004
	Proteobacteria	Deltaproteobacteria	Desulfarculales	0.04	0.04	0.03	0.03											0.0036	0.0031	0.0001
			Desulfobacterales	0.20	0.02	0.01		0.25	0.12	0.21	0.12			0.0006		0.0001	0.0009	0.0086	0.0011	0.0002
			Desulfuromonadales					0.01						0.0006						
			Other	0.05	0.03	0.03	0.02					0.0044				0.0043		0.0032	0.0058	
		Gammaproteobacteria							0.04				0.0003	0.0005		0.0004	0.0002		0.0002	0.0004
	Spirochaetes	Spirochaetia	Spirochaetales	0.02	0.01	0.02	0.01										0.0002	0.0022	0.0003	0.0013
	WS1													6.194E-05				0.0008		
	WS2																	0.0036		
	Zixibacteria				0.01	0.02	0.03		0.01							0.0007	0.0011	0.0015		

Table S2, Recipe for f/2 media used for the growth of algal biomass used as substrate in the PM and protein amended slurries.

Recipe for 1 liter of: f/2 medium + 4 mM $NaHCO_3$

Component	mw			working stock quantity (g)	working stock mM	quantity (ml)	final conc (µM)
$NaNO_3$	84.99			57.7	679	1.3	883
$NaH_2PO_4*12H_2O$	358.14			9.9	28	1.3	36
$NaHCO_3$	84.007			34	405	10	4047
trace metal solution						1.3	
f/2 vitamin solution						1	
Aged seawater (filtered 0.2 µM)						1 L	

trace metal solution	mw	primary stock g/100 ml	primary stock mM	working stock quantity (g)	working stock mM		final conc (µM)
$FeCl_3·6H_2O$	270.3			2.42	9.0		11.64
$Na_2EDTA·2H_2O$	374.24			3.35	9.0		11.64
$CuSO_4·7H_2O$	285.72	0.08	2.8	10 ml	0.03		0.04
$ZnSO_4·7H_2O$	287.56	0.17	5.9	10 ml	0.06		0.08
$CoCl_2·6H_2O$	237.93	0.08	3.4	10 ml	0.03		0.04
$MnCl_2·4H_2O$	197.91	1.4	70.7	10 ml	0.71		0.92
$Na_2MoO_4·2H_2O$	241.95	0.17	7.0	10 ml	0.07		0.09

f/2 vitamin solution		primary stock g/10 ml	primary stock mM	working stock quantity (ml)	working stock mM		final conc (µM)
Biotin	244.31	0.005	2.04658	1	0.002047		0.0020
Thiamine-HCl	337.3	1	296.472	1	0.296472		0.2965
Cyanocobalamin	1335.38	0.005	0.374425	1	0.000374		0.0004

Added all sterilized stock solutions to autoclaved aged seawater to a total of 1 L volume. Stored the f/2 medium for at least 24 h before use in the dark at the growth temperature of the algae to be cultured. For labelled cultures replaced 30% ^{13}C-$NaHCO_3$ and 10% ^{15}N-$NaNO_3$.

Table S3, Recipe for the minimal BA brackish water media used as liquid phase in sediment slurries

BA brackish water media salts
according to the CCY collection, where MgSO4 replaced by additional MgCl2

	mM	mg/l
**$NaHCO_3$**	3	168.0
**KCl**	2.14	159.5
**NaCl**	142	8298.5
**$MgCl_2 \cdot 6H_2O$**	7.9	1606.1
**$CaCl_2 \cdot 2H_2O$**	0.99	145.5
	salinity:	10377.6
**resazurin**	0.25 mg/l	from stock
**Na_2S max**	0.085	6.63
